# Genome-wide analysis of WRKY transcription factors involved in abiotic stress in *Lonicera japonica*

**DOI:** 10.3389/fpls.2025.1653750

**Published:** 2025-08-14

**Authors:** Zhihui Li, Bing Pi, Sisi Liu, Yongxin Li, Neng Cai, Jiqing Peng, Chong Liu, Zhongquan Qiao

**Affiliations:** ^1^ Hunan Key Laboratory for Breeding of Clonally Propagated Forest Trees, Hunan Academy of Forestry, Changsha, Hunan, China; ^2^ Yuelushan Laboratory for College of Life and Environmental Sciences, Central South University of Forestry and Technology, Changsha, Hunan, China; ^3^ Changsha Environmental Protection Vocational College, Hunan, China

**Keywords:** WRKY, *Lonicera japonica*, medicinal plant, abiotic stress, expression analysis, bioinformatics

## Abstract

The WRKY transcription factor family, one of the largest gene families in plants, plays crucial roles in regulating growth, stress responses, and environmental adaptation. However, the specific functions and regulatory mechanisms of *WRKY* genes in *Lonicera japonica* (honeysuckle) under drought and salt stress remain poorly characterized. In this study we identified 41 *LjWRKY* genes from the *L. japonica* genome. These genes are unevenly distributed across nine chromosomes. Phylogenetic analysis based on the conserved WRKY domain classified the LjWRKYs into Groups I, II, and III. Promoter analysis revealed an abundance of light-responsive elements, hormone-related elements and abiotic stress-related elements within the *LjWRKY* genes. Analysis of gene duplication events identified 70 gene pairs under strong purifying selection during evolution. Notably, comparisons with *Lonicera macranthoides* revealed 5 genes exhibiting exceptionally strong conservation (Ka/Ks < 0.1), suggesting potential roles as housekeeping genes. Two *LjWRKY* genes (*LjWRKY22* and *31*) were identified as key regulators through correlated expression patterns with stress-responsive physiological biomarkers. This study elucidates key regulatory mechanisms of LjWRKY transcription factors in *L. japonica* ‘s response to drought and salt stress. Our findings provide specific candidate genes for further investigation into *WRKY* functional evolution and offer a molecular basis for developing enhanced drought- and salt-tolerant *L. Japonica* cultivars.

## 1 Introduction

Plant growth is frequently limited by abiotic stresses, with drought, and soil salinization posing particularly significant threats. To cope with these challenges, plants have evolved complex stress response mechanisms. Drought stress, caused by water scarcity, severely impairs plant growth, development, and yield, leading to detrimental effects such as the accumulation of reactive oxygen species (ROS) (Yang et al., 2021). Similarly, soil salinization is a major environmental constraint that adversely affects plant growth, agricultural productivity, and ecological stability (Hasegawa et al., 2000; Zhu, 2003). Therefore, developing innovative and sustainable strategies to enhance plant tolerance to drought and salinity is imperative.

The WRKY transcription factor family represents one of the largest transcription factor families in plants and plays a crucial role in growth, development, and, importantly, responses to abiotic stresses (Eulgem and Somssich, 2007; Chen et al., 2012; Li et al., 2020). A defining feature of WRKY proteins is the conserved WRKY domain, containing the WRKYGQK motif that recognizes and binds the W-box cis-element in target gene promoterse (Maleck et al., 2000; Lee et al., 2009; Ishihama and Yoshioka, 2012; Machens et al., 2013). Based on the number and type of WRKY domains, they are classified into three major groups (I, II, III).

WRKY transcription factors are an integral part of signal transduction and gene expression regulation during biotic and abiotic stress responses. Numerous studies across diverse plant species have demonstrated that WRKY TFs play pivotal roles in plant adaptation to drought and salinity stresses, though their functions can be either positive or negative regulators of tolerance. For instance, *AtWRKY1* in *Arabidopsis thaliana* positively regulates drought tolerance by modulating ABA signaling and stomatal responses (Qiao et al., 2016). Similarly, overexpression of soybean *GmWRKY16* enhances tolerance to osmotic and salt stress in *A. thaliana* (Ma et al., 2019). Conversely, rice *OsWRKY5* and *OsWRKY114* act as negative regulators of drought stress tolerance (Lim et al., 2022; Song et al., 2022). However, despite the well-established importance of WRKY TFs in plant stress adaptation, their specific functions and regulatory mechanisms in the important medicinal plant *Lonicera japonica* (honeysuckle), particularly in response to drought and salt stress, remain largely unexplored.

*Lonicera japonica* Thunb, (honeysuckle or Jinyinhua), a semi-evergreen climbing vine of the Caprifoliaceae family native to East Asia, holds significant medicinal and economic value. However, its cultivation is constrained by limited tolerance to drought and salinity, restricting its geographic distribution and full potential utilization. Therefore, identifying genes conferring stress tolerances and elucidating the expression patterns under adverse stress conditions are crucial steps for breeding resilient varieties. However, current research on the LjWRKY transcription factor family remains significantly limited. A comprehensive genome-wide identification and systematic analysis is lacking, and their specific functions and regulatory networks in response to drought and salt stress remain poorly characterized. Elucidating the *LjWRKY*-mediated stress resistance mechanisms is crucial for understanding *L. japonica’*s environmental adaptation, mining key genetic resources for stress tolerance, and guiding molecular breeding efforts.

In this study, we systematically identified and characterized a comprehensive repertoire of 41 WRKY transcription factor within the *L. japonica* genome. We conducted a thorough analysis of their phylogenetic relationships, conserved motifs, gene structures, chromosomal distributions, and evolutionary history, identifying gene duplication events under strong purifying selection. Employing quantitative reverse transcription polymerase chain reaction (qRT-PCR), we elucidated the expression dynamics of nine selected *LjWRKY* genes (*LjWRKY13, 16, 17, 29, 41* under drought; *LjWRKY10, 22, 31, 36* under salt stress) across tissues and developmental stages in response to drought and salt stress. The findings from this study provide the foundational genomic resource for the *LjWRKY* family and identify key candidate genes involved in stress adaptation, establishing a strong framework for future functional characterization of *LjWRKYs* in mediating *L. japonica*’s resilience to drought and salinity.

## 2 Materials and methods

### 2.1 Plant materials, growth conditions, and stress treatments

Samples of the *L. japonica* variety “Fenglei” were collected from the experimental base in the experimental forest of the Hunan Academy of Forestry. Thirty-six uniformly growing, one-year-old cutting-propagated *L. japonica* plants(height: 60–80 cm; basal diameter: 5.8-7.1 mm) were selected and randomly allocated to two groups of eighteen plants each. These plants were matched according to growth status, size, and height. Under a constant temperature of 25°C, with 16 hours of light and 8 hours of darkness. In the salt stress treatment, 50 ml of 400 mM NaCl solution was added to each pot every day for five consecutive days. The sampling days were labeled as D1 to D5. The control group is D0 without any NaCl treatment. In the drought stress treatment, samples were collected at 5-day intervals starting from day 20 after irrigation cessation, with these time points designated as D1 to D5. Throughout the experimental period, control group (D0) plants were irrigated with 300 mL of water per pot at 48-hour intervals. Mature leaves, stems, and roots were harvested from actively growing *Lonicera japonica* plants during vegetative growth stage, and three biological replicates were performed at each time point. Immediately after collection, all samples were quickly frozen in liquid nitrogen to preserve their integrity and then stored at -80°C.

### 2.2 Genome-wide identification and distribution of *LjWRKYs*

To thoroughly identify the *LjWRKY* gene family of *L. japonica*, this study utilized the whole-genome sequence data provided on the website of the National Bioinformatics Center (https://ngdc.cncb.ac.cn/gwh/Assembly/660/show/, accessed on April 17, 2023). The Hidden Markov Model (HMM) profile of the WRKY domain (number PF03106) is from the Pfam database (https://www.ebi.ac.uk/interpro/entry/pfam/, accessed on April 13, 2024). Through the hmmer 3.0 software suite, the hmmsearch command was used to search for potential *WRKY* genes. A strict criterion of e-value < 1×10^-5^ was applied to screen the search results, obtaining an initial set of 46 *LjWRKY* genes. After preliminary screening, the genes were analyzed in detail using NCBI cdsearch tool to confirm the presence of conserved WRKYGQK motifs and C2H2/C2HC zinc finger structures. This process excluded five genes, possibly due to inaccurate sequencing, such as gene splicing errors. The remaining 41 genes were systematically renamed as *LjWRKY1* to *LjWRKY41* according to their chromosomal order. The protein parameter calculator tool in the TBtools software suite was used to further characterize the LjWRKY protein. This analysis includes determining the amino acid composition, molecular weight, isoelectric point, and instability index of each LjWRKY protein (Chen et al., 2023). The online website WoLF PSPRT (https://wolfpsort.hgc.jp/, accessed on April 17, 2023) was used to predict the subcellular localization of 41 LjWRKY protein based on K-Nearest Neighbors (k-NN) and sequence features.

### 2.3 Phylogenetic analysis of *WRKY* genes

The *Arabidopsis thaliana* Information Resource Database (TAIR) (https://www.arabidopsis.org/, accessed on April 13, 2024) was used to obtain 71 *WRKY* genes in *A. thaliana*. Focusing on the conserved WRKY domain in these genes, a phylogenetic tree including *L. japonica* and *A. thaliana* was constructed. Using MEGA11 software, Using MEGA11 software, the best-fit substitution model was first determined through the “Find Best DNA/Protein Models” function based on the Bayesian Information Criterion (BIC). Subsequently, a phylogenetic tree was constructed employing the Jone-Taylor-thoronton (JTT) model and Neighbor-Joining (NJ) method, with bootstrap values calculated from 1000 replicates. The Chiplot online tool (https://www.chiplot.online/, accessed on April 15, 2024) was used for visualization (Xie et al., 2023). Multiple sequence alignment was carried out using the Espript3.0 online website (https://espript.ibcp.fr/ESPript/cgi-bin/ESPript.cgi, accessed on April 16, 2024).

### 2.4 Analysis of conserved motifs and domains

The MEME online website (http://meme-suite.org, accessed on April 13, 2024) was used, setting the limit to 10 motifs with a length range of 6 to 50 amino acids to characterize the conserved motifs in WRKY proteins (Bailey et al., 2009). In addition, the NCBI Conserved Domain Database (CDD) (https://www.ncbi.nlm.nih.gov/Structure/cdd/wrpsb.cgi, accessed on April 14, 2024) was used to check for the presence of the WRKY domain in these proteins. The conserved motifs and domains were visualized with the help of the Chiplot tool (https://www.chiplot.online/, accessed on April 15, 2024).

### 2.5 Promoter sequence analysis

The Tbtools software was used to extract the sequence file of the upstream 2000bp region of the promoter. The online website PLANTCARE (http://bioinformatics.psb.ugent/web/webtools/plantcare/html/, accessed on May 23, 2024) was used to analyze the regulatory elements in the promoter sequence of *WRKY* genes. These elements were visualized using Chiplot (https://www.chiplot.online/, accessed on May 24, 2024).

### 2.6 Chromosome localization and collinearity analysis

The TBtools software was used to study the distribution of *LjWRKY* genes on chromosomes, and its OnestepMCscanx module is used to analyze the mechanisms of gene family expansion, which primarily involves tandem and dispersed repeat expansions. The results were visualized with the Circos tool of TBtools. To explore homology relationships, the whole genome was downloaded from the website of the National Bioinformatics Center (https://ngdc.cncb.ac.cn/gwh/Assembly/660/show/, accessed on April 17, 2023), and visualized using the Dual system Plot module of TBtools (Chen et al., 2023). The calculation of evolutionary pressure was performed using Tbtools software. For molecular clock estimation, the formula T=Ks/(2×μ) was applied, where the μ value was set as 1.5×10^-8^, referencing the parameter reported in *A. thaliana* (Bennetzen et al., 2005). The network visualization and topology analysis were completed by Cytoscape 3.9.1, the hub node screening was performed by NetworkAnalyzer tool, and the statistical significance was determined by comparing with the random network (Erdos-Renyi model, 1000 times repeated, P<0.01).

### 2.7 Expression pattern analysis

Total RNA was extracted using the EASYspin Plus Complex Plant RNA Kit (Aidlab, Beijing, China), and reverse transcribed into cDNA according to the cDNA synthesis kit (Vazyme, Jiangsu, China). qRT-PCR primers were used, and the primer list is shown in **Supplementary Table S1**. qRT-PCR was performed using QuantStudio™ Real-Time PCR software (Version 1.3), and *LjACT7* was used as an internal reference gene (Livak and Schmittgen, 2001). To ensure accuracy, a total of three biological replicates and three technical replicates were conducted, and gene expression was calculated using 2^−ΔΔCT^ (Livak and Schmittgen, 2001).

### 2.8 Determination of physiological and biochemical indicators

Leaves under drought and salt stress from D0 to D5 were collected for physiological and biochemical analysis. Four physiological and biochemical indicators including catalase, peroxidase, malondialdehyde, and proline were analyzed using the Solarbio kit (Beijing, China).

### 2.9 Statistical analysis

SPSS27 software was used to analyze the correlation between physiological indicators and gene expression under stress conditions. A heat map was created using ChiPlot (https://www.chiplot.online/, accessed on July 9, 2024) to visualize these correlation relationships.

## 3 Results

### 3.1 Identification and characterization of the *LjWRKY* gene family

In this study, 41 genes belonging to the *WRKY* family of *L. japonica* were identified. They were named *LjWRKY1* to *LjWRKY41* according to their order on the chromosome. The summary of their physicochemical properties is presented in **Supplementary Table S2**. The sizes of LjWRKY proteins vary. LjWRKY40 is the smallest with 200 amino acids, while LjWRKY36 is the largest with 748 amino acids. The molecular weight of LjWRKY40 is approximately 21.93 kDa, and that of LjWRKY36 is about 80.97 kDa. The isoelectric point represents the pH value at which a protein has no net charge. LjWRKY14 has an isoelectric point of 4.85, and LjWRKY38 has 9.71, with an average of 7.06. The aliphatic index, which measures protein stability in the environment, ranges from 41.00 for LjWRKY40 to 71.05 for LjWRKY30. The instability index is higher than 40 for all proteins, indicating their instability. This suggests that their primary structure is the main factor affecting their variability in the laboratory. The total average hydrophilicity of all LjWRKY proteins is negative, a sign of their hydrophilic nature, implying that they may exist and function intracellularly. Regarding their cellular localization, predictions show that almost all proteins are localized in the nucleus except for LjWRKY28, which is predicted to be in the chloroplast.

### 3.2 Grouping and interrelationships of *LjWRKY* genes

**Figure 1:**
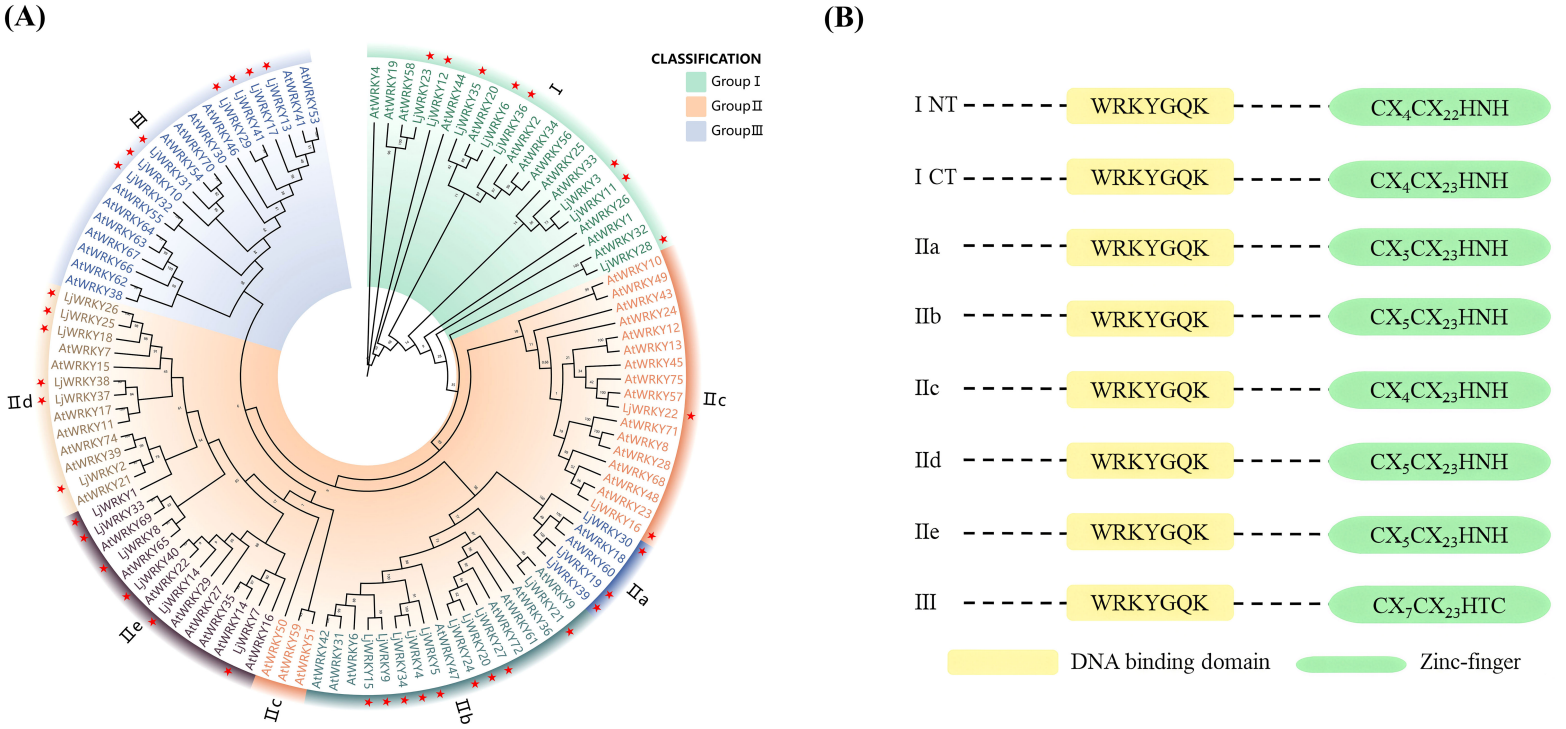
**(A)** Phylogenetic tree of WRKY proteins from *L. japonica* and *A*. *thaliana*. “At” represents *A*. *thaliana* and “Lj” represents *L. japonica*. The leaves marked with a star are *L. japonica*. **(B)** Domain structure of different WRKY subfamilies in plants. ICT and INT indicate domains at the N-and C-termini of group I WRKY proteins, respectively (Eulgem et al., 2000).

**Figure 2:**
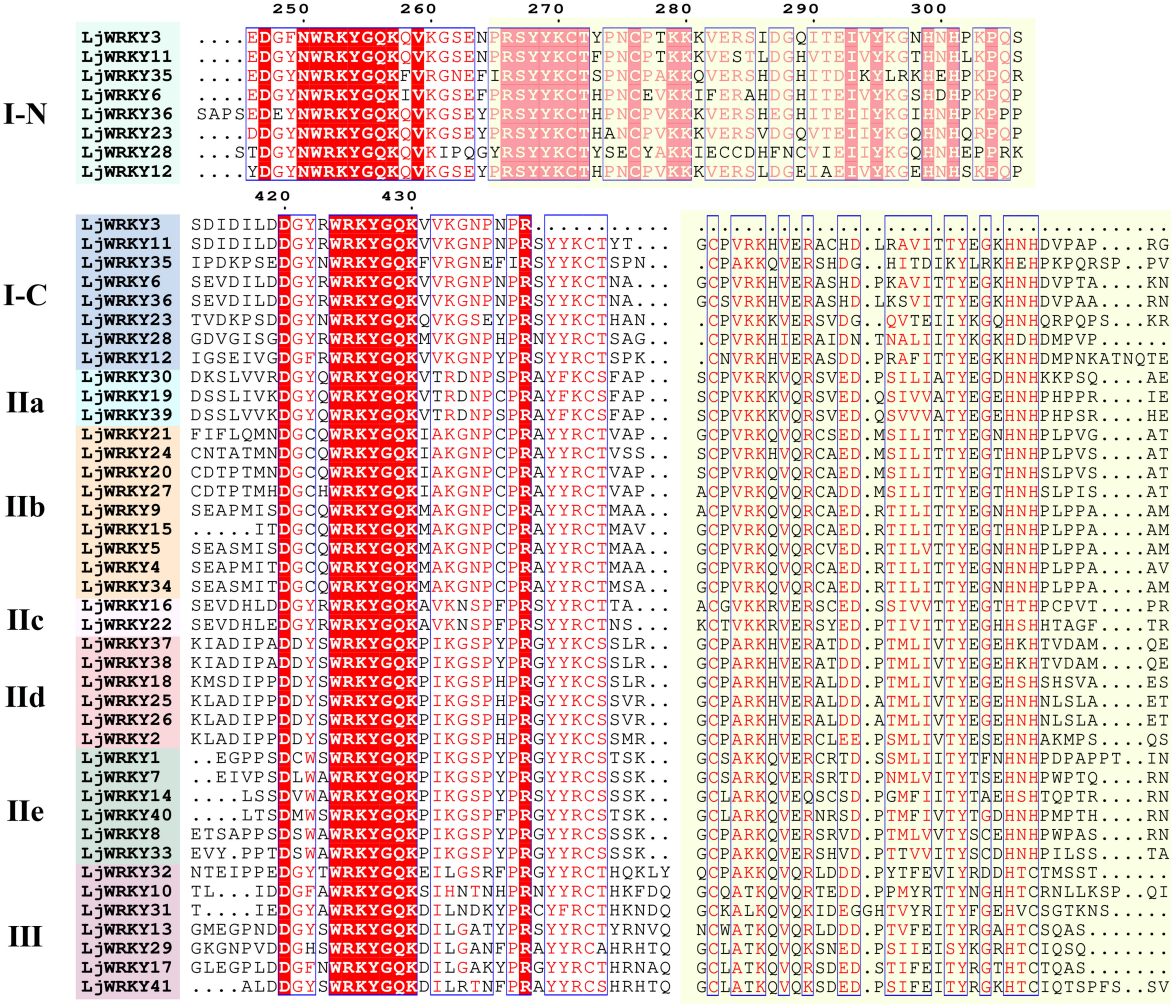
Multiple sequence alignment of LjWRKYs. The conserved WRKY amino acid sequence is filled in red, and the zinc finger structure is filled in yellow. Other conserved WRKY amino acid sequences are marked in blue boxes. “N” and “C” represent the N-terminal and C-terminal WRKY domains of group I, respectively.

Based on the protein sequences of *WRKY* genes from *L. japonica* and *A. thaliana*, a phylogenetic tree was created using MEGA11 (**Figure 1A**). According to the WRKY domain and zinc finger structure characteristics of the N and C ends of WRKY transcription factor, WRKY transcription factor is divided into three subfamilies: The second subfamily is further subdivided into five subgroups, IIa, IIb, IIc, IId and IIe, according to the sequence characteristics of the conserved domain (**Figure 1B**). It was found that the 41 *LjWRKY* genes can be divided into three major categories: the first category has 8 members, the second category has 26 members, and the third category has 7 members. Within the second category, it is further divided into five subgroups named II-a (3 members), II-b (9 members), II-c (2 members), II-d (6 members), and II-e (6 members) (**Figure 1**). In the evolutionary tree, II-a, II-b, II-d, and II-e are closely related. Interestingly, group II-c seems to be more closely related to group I. Based on the protein sequences of the LjWRKY family, a multiple sequence alignment was performed. The alignment results are shown in **Figure 2**. All LjWRKYs have a WRKY motif containing a core region of seven amino acids (WRKYGQK). However, it is noteworthy that the zinc finger structure of some genes has a single base substitution, which may impact the function of these genes. For example, LjWRKY3 loses a zinc finger structure at its C-terminus.

### 3.3 Analysis of conserved motifs, domains, and gene structures in the *LjWRKY* gene family

**Figure 3:**
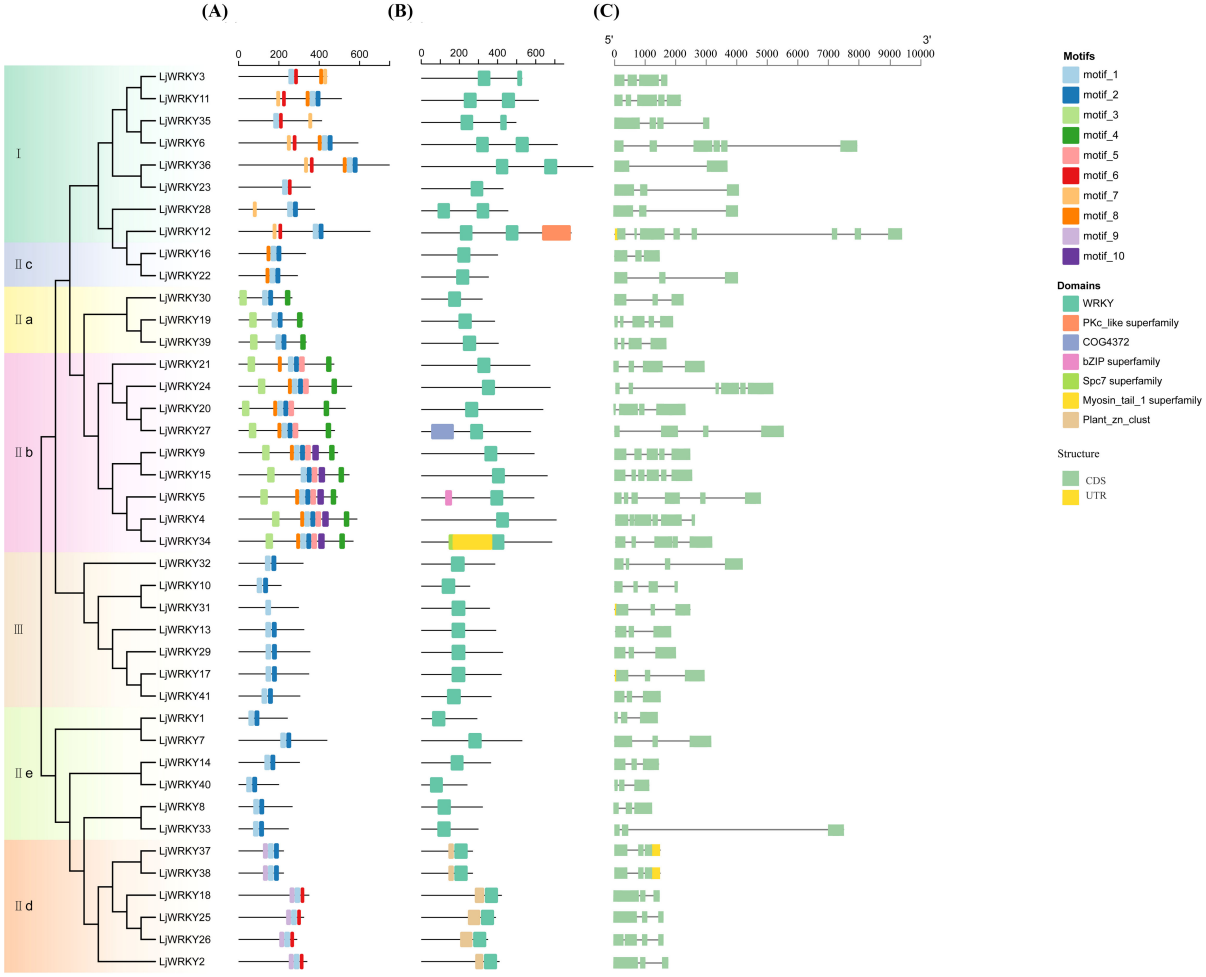
Motif distribution and conserved domain analysis of LjWRKYs. **(A)** Conserved motifs of LjWRKY. **(B)** Conserved domains of LjWRKY. **(C)** Gene structure analysis.

The online website MEME was used to predict 10 motifs of 41 LjWRKY proteins, labeled Motif1 to Motif10. The sequence lengths of the 10 motifs range from at least 19 amino acids (Motif 6) to at most 38 amino acids (Motif 3). Motif 1 and Motif 7 contain the typical conserved heptapeptide of the WRKY domain, and Motif 2 is the zinc finger motif. From the motif distribution characteristics of members of each subfamily in the evolutionary tree, it can be clearly seen that genes within the same subfamily show similar motif compositions, but there are obvious differences between subfamilies (**Figure 3A**). This indicates that the motif pattern may be related to the function of LjWRKY proteins. Some motifs are conserved among family members. For example, Motif 1 and Motif 2 are present in almost all protein sequences, indicating that these motifs are the most important components of LjWRKY proteins. Some other motifs only exist in some members. For example, Motif 10 is found in 12.20% of LjWRKY members, and Motif 9 is present in 14.63% of LjWRKY members. The motif analysis of LjWRKY proteins shows that members under the same subfamily are highly conserved, further proving the proximity of genes in phylogenetic evolutionary relationships. In addition, the motif composition of members of group IIe is highly similar to that of group III. Judging from the evolutionary tree, their evolutionary distance is also closer. LjWRKY31 of group III only contains one Motif type, which may suggest that this protein has a unique function.

The NCBI Conserved Domain Database (CDD) was used to predict the conserved domains of 41 LjWRKY proteins. It was found that all LjWRKYs have a WRKY domain (**Figure 3B**). Combined with the evolutionary tree of the LjWRKY family, it can be seen that members of group IId all have a Plant_zn_clust domain, which may be related to the different functions of this subfamily. In addition, some individual proteins contain special domains. For example, LjWRKY12 contains a Pkc_like domain, which has been proven to be able to control gene expression initiation and repression, transfer proteins, and regulate cell growth (Miranda-Saavedra and Barton, 2007). LjWRKY34 not only has a unique domain of WRKY but also contains two special domains, namely Spc7 and Myosin_tail_1 domains. Spc7 is closely related to cell division (Kerres et al., 2004), suggesting that LjWRKY34 may play a role in cell division.

In the gene structure diagram (**Figure 3C**), only five genes (*LjWRKY12, 17, 31, 37, 38*) contain untranslated regions (UTRs), distributed across Group I, IId, and III. The sparse distribution of UTRs (12.2% of total *WRKY* genes) may reflects evolutionary specialization in *L. japonica WRKY* regulation: while most members achieve rapid stress response through protein-protein interactions (e.g., hub networks formed by *LjWRKY9/34/15*), UTR-containing genes likely employ post-transcriptional mechanisms (such as providing miRNA target sites) for precise expression control. It should be noted that the detection of UTRs is highly dependent on the completeness of genome annotation. The absence of observed UTRs in some *WRKY* genes is likely due to incomplete coverage of these regions in the current annotation.

### 3.4 Promoter sequence analysis of the *LjWRKY* gene family

**Figure 4:**
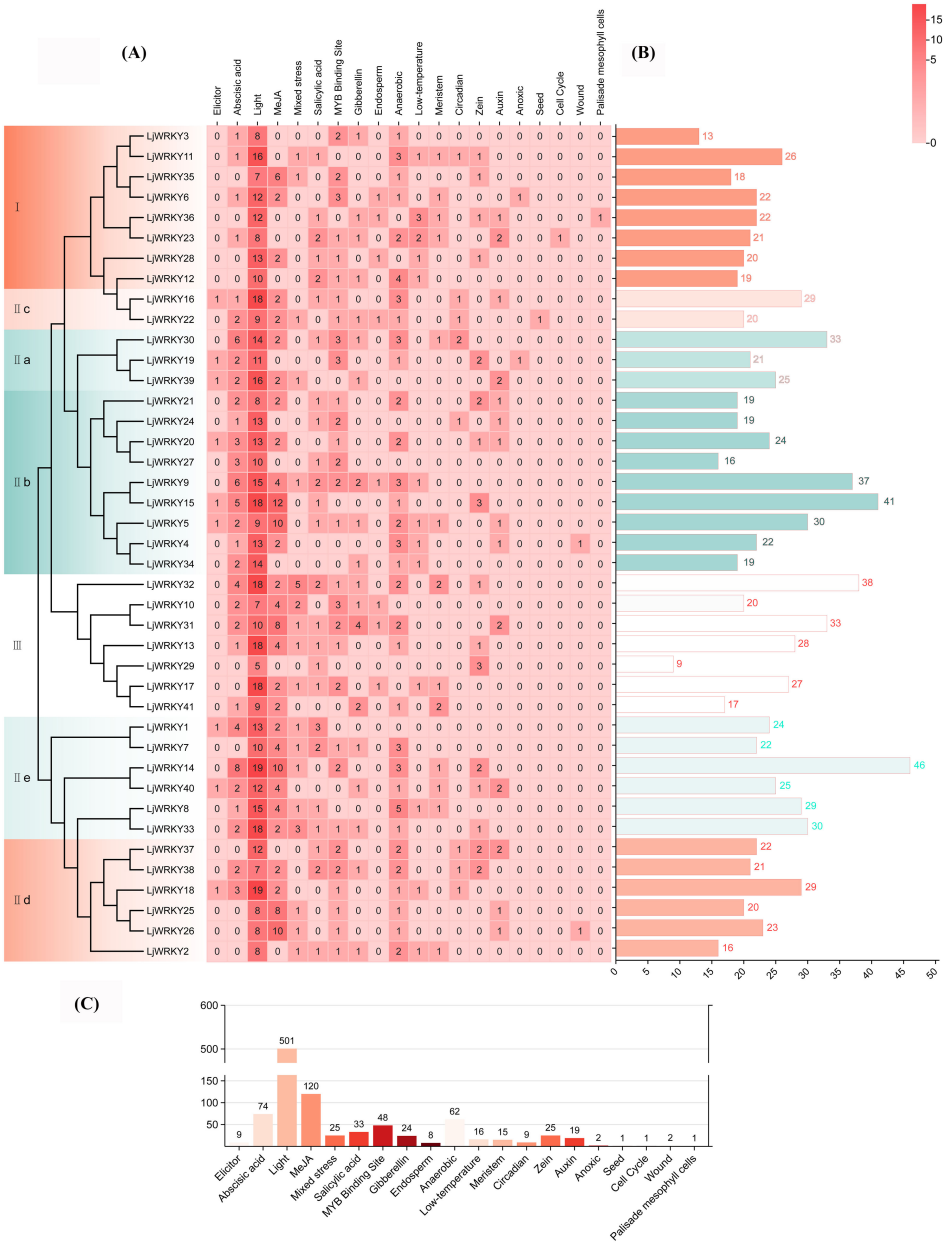
Promoter sequence analysis of *LjWRKYs*. **(A)** Clustering heat map of cis-acting elements. The numbers in the Figure represent the number of transcription factor binding sites. The darker the color, the greater the number. **(B)** Histogram of the number distribution of cis-acting elements of *LjWRKYs*. **(C)** Histogram of the number distribution of types of cis-acting elements.

The Plantcare website was used to obtain information on cis-acting elements in the first 2000 base pairs of the start region of the *LjWRKY* gene, including light-responsive elements (such as G-box, GT1-motif and Sp1), elements responsive to the plant hormone MeJA (with TGACG and CGTCA motifs), elements responsive to the plant hormone abscisic acid (ABRE), elements responsive to cold temperature (LTR), elements responsive to the plant hormone auxin (TGA-element and AuxRR-core), and elements responsive to the plant hormone gibberellin (TATC-box) and other 20 elements (**Figure 4**). Among these 20 elements, light-responsive elements and methyl jasmonate-responsive elements account for the largest proportions, 50.84% and 12.12% respectively. Followed by abscisic acid-responsive elements, which account for 6.21% of all elements. Each gene contains from 9 (*LjWRKY29*) to 46 (*LjWRKY14*) elements, and the number of types is 3 to 10. The diversity of promoter cis-acting elements predicts the richness of gene functions. Light-responsive elements are distributed in most *LjWRKY* genes. Among them, *LjWRKY14* and *LjWRKY18* have a large number of light-responsive elements. Different *LjWRKY* genes contain different types, numbers, and positions of promoter elements, indicating that *LjWRKY* genes jointly participate in responding to various abiotic stresses through multiple promoter cis-acting elements to complete plant growth and development.

### 3.5 Chromosome localization and correlation study of *LjWRKY* genes

**Figure 5:**
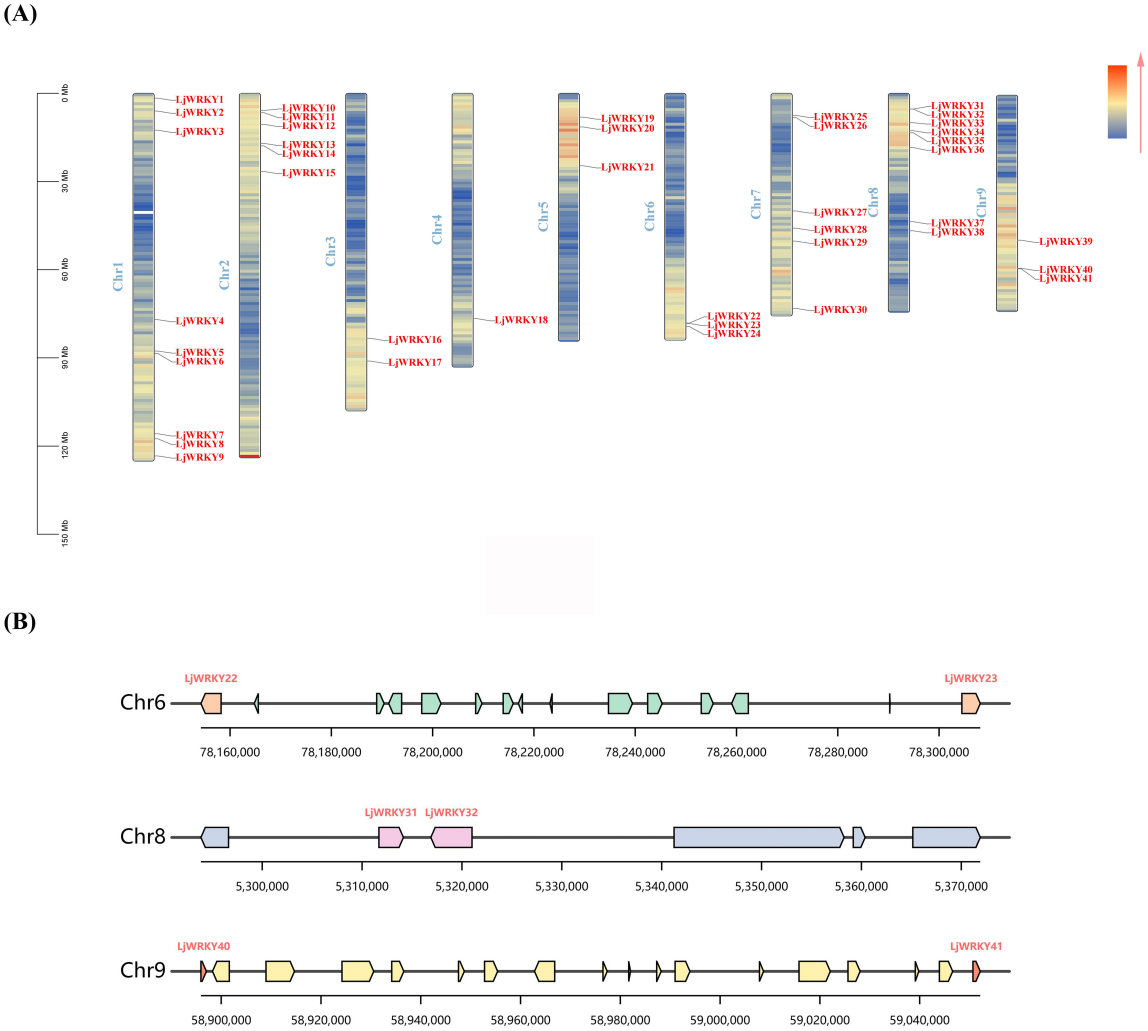
**(A)** Chromosome location of *LjWRKY*s **(B)** Genomic environment of the three potential *LjWRKY* gene clusters. The background color gradient represents the density of genes.

**Figure 6:**
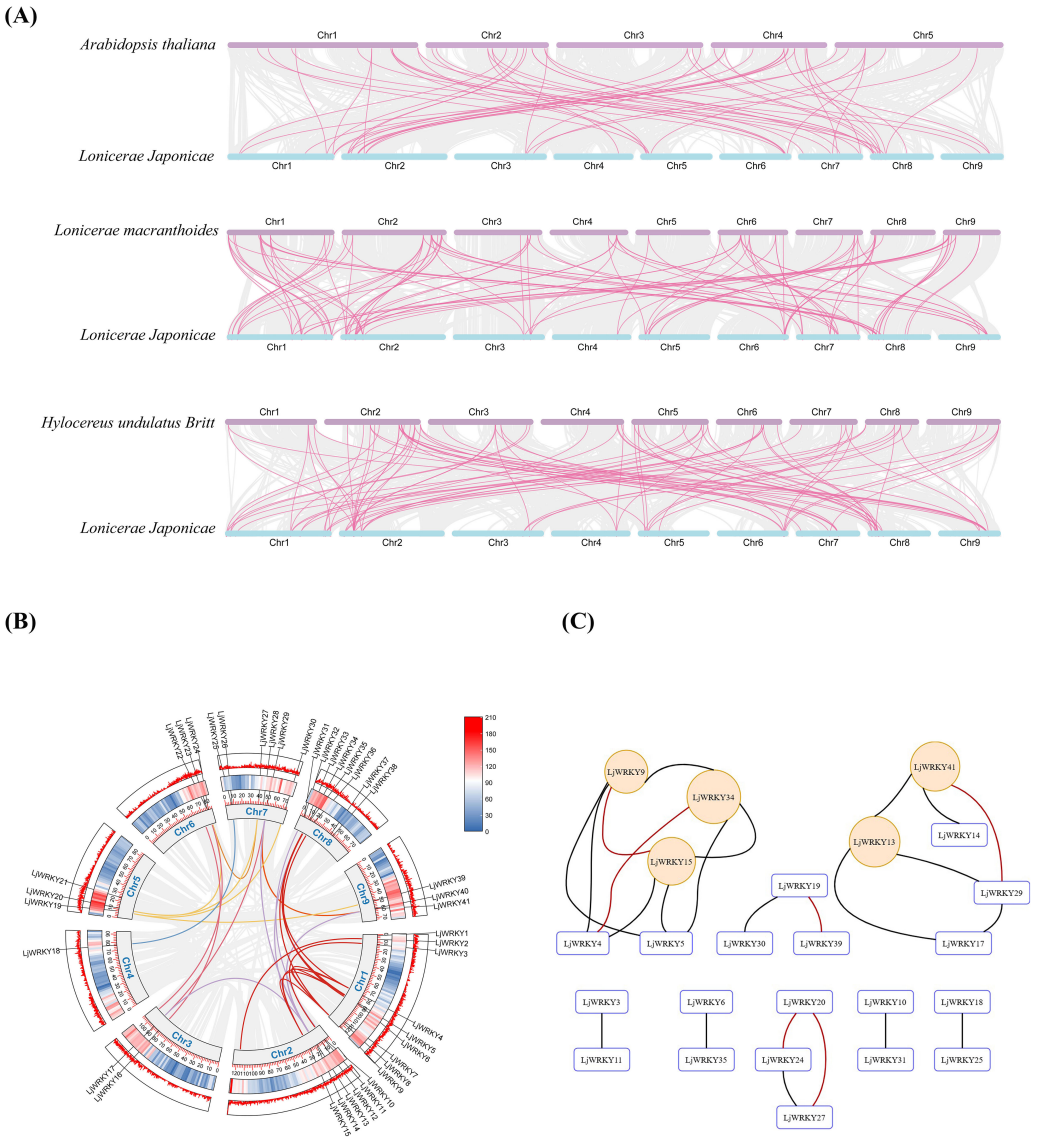
**(A)** *L. japonica* and three representative plant species. The gray line in the background represents all the collinear relationships between the genomes of *L. japonica* and other plants. The pink line highlights the similar gene pairs of *LjWRKY*. **(B)** Isomorphic analysis of the relationship between *LjWRKYs* chromosomes. The gray line represents all the homologous blocks in the genome of *L. japonica*. Different lines represent different chromosomes. **(C)** Visualization of the evolutionary relationship network within the *L. japonica* WRKY family. Hub nodes are highlighted in orange (identified by Erdős–Rényi model with 1000 replicates, P<0.01), and pairs under strong purifying selection are denoted by thick red edges (Ks<1.5 and Ka/Ks<0.3).

Chromosome localization shows that 41 *LjWRKY* genes are unevenly distributed on 9 chromosomes. The density on chromosome 1 is the highest, with 9 genes, while the density on chromosome 4 is the lowest, with only 1 gene (**Figure 5A**). Chromosome mapping indicated that the *LjWRKY* family members potentially form clusters on three chromosomes. Visualization of these putative cluster regions (**Figure 5B**) revealed that only *LjWRKY31* and *LjWRKY32* on chromosome 8 are closely linked, with no other genes intervening. This arrangement is inferred to result from a tandem duplication. In contrast, the other *LjWRKY* genes appear to have arisen from either segmental duplications or dispersed duplications, based on their respective genomic locations. TBtools was used for collinearity analysis within the *L. japonica* species to study the expansion mechanism of the WRKY transcription factor gene family of *L. japonica*. Collinearity analysis is mainly used to predict gene duplication events, including tandem duplication and segmental duplication (Bennetzen et al., 2005) (**Figure 6A**). Based on the circos diagram, a total of 31 pairs of collinear genes were found (**Figure 6B**), some genes are not *WRKY* genes (Shown in **Supplementary Table S5**), which may be the result of fragment duplication. Changes in certain specific domains during evolution may lead to great changes in function. Network visualization of *L. japonica WRKY* gene pairwise relationships was performed (**Figure 6C**), identifying five hub nodes based on degree and betweenness centrality (**Supplementary Table S5**). These evolutionarily conserved hubs (e.g., *LjWRKY9* and *LjWRKY41*) may function as molecular switches that coordinate stress responses by integrating multiple signaling inputs. Their functional redundancy (exemplified by co-regulation between *LjWRKY34* and *LjWRKY9*) provides critical targets for crop resistance breeding. To further explore the relationship between the *LjWRKY* genes of *L. japonica* and those of other species, this study compared the chromosomal locations of *L. japonica* with *A. thaliana*, *L. macranthoides*, and *Hylocereus undulatus Britt*. There are 41 pairs of *LjWRKY* genes in *Lonicera macranthoides*, followed by *Hylocereus undulatus Britt* with 35 pairs, and *A. thaliana* with 33 pairs. Among them, 29 *LjWRKY* genes show obvious patterns of kinship in all three species (**Supplementary Table S5**). Interestingly, some *LjWRKY* genes have multiple corresponding genes in other species, indicating a complex history of gene duplication and differentiation.

By analyzing non-synonymous and synonymous substitutions in these gene pairs, the Ka/Ks values and divergence times for each pair were calculated. The results are presented in **Supplementary Table S5**. After excluding gene pairs with Ks> 1.5 and those that were calculated to be saturated, all strongly purged selection gene pairs were selected based on Ka/Ks <0.3. There are 10 gene pairs within the *L. japonica* gene family, 1 pair between *L. japonica* and *A. thaliana*, 17 pairs between *L. japonica* and *Hylocereus undulatus Britt*, and 41 pairs of strongly purged selection gene pairs between *L. japonica* and its close relative, *L. macranthoides*. Additionally, 5 pairs of gene pairs with Ka/Ks <0.1 were observed. They are the genes corresponding to *LjWRKY2,3,14,18,26* and *Lonicera macranthoides* Genes, which means that these five genes are extremely conserved and are likely to be housekeeping genes. Furthermore, by referencing the mutation rate of *A. thaliana*, the divergence times of these gene pairs were calculated, which reflects the evolutionary rate of molecular clocks. A short molecular clock indicates that a specific DNA or protein sequence accumulates mutations at a rapid rate during evolution, suggesting significant changes over a given geological time span. Among these gene pairs, the shortest divergence time is 450,000 years, while the longest is 49 million years, and they are found between *L. japonica (LjWRKY2)* and *L. macranthoides*, as well as *L. japonica (LjWRKY39)*and *Hylocereus undulatus Britt.*

### 3.6 Response of *Lonicera japonica* to environmental stress

**Figure 7:**
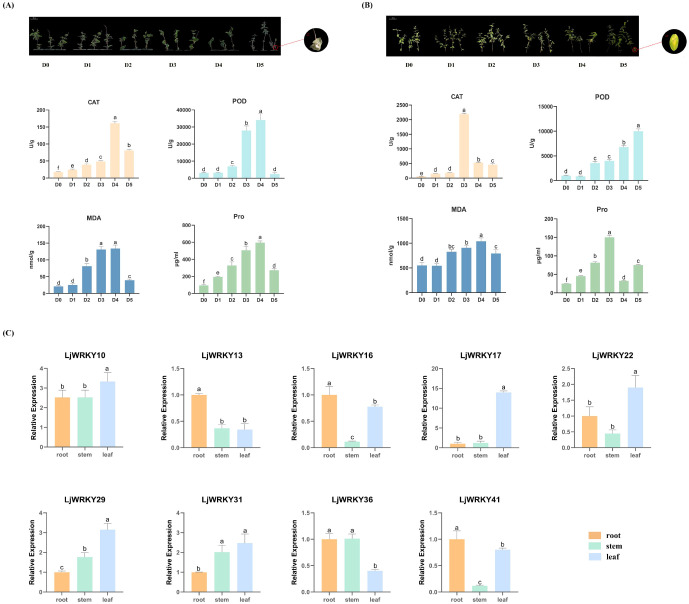
**(A)** Phenotypic progression and physiological dynamics during drought stress. D0 (Control, well-watered); D1-D5 (Days 21–25 post-watering withdrawal). Inset: Leaf morphology at D5; Bottom: Changes in catalase (CAT), peroxidase (POD), malondialdehyde (MDA), and proline (Pro) contents over D0 and D1-D5. **(B)** Phenotypic progression and physiological dynamics during salt stress. D0 (Control, untreated); D1-D5 (Days 1–5 of consecutive NaCl treatment); Inset: Leaf morphology at D5; Bottom: CAT, POD, MDA, and Pro contents across D1-D5 salt exposure. **(C)** Tissue-specific expression profiles of nine *LjWRKY* genes under control conditions. RT-qPCR analysis of target genes in roots, stems, and leaves at D25 (well-watered control). Significance: Bars labeled with different lowercase letters a-f indicate statistically distinct values (p< 0.05; “a” = highest value).

*L. japonica* exhibited distinct leaf phenotypic progression under drought versus salt stress based on visual observation. Drought triggered sequential alterations: initial loss of turgor manifested as softened, dark-green leaves (D1-D3), progressing to inward curling (D4), and culminating in brittle necrosis (D5). However, throughout the duration of salt stress exposure, honeysuckle exhibited progressive yellowing and withering of leaves, culminating in near-complete yellowing of D5 foliage by the experimental endpoint. These morphological alterations corresponded with physiological indicator fluctuations. During drought stress, CAT, POD, MDA, and proline simultaneously peaked at D4 (p<0.05) before declining sharply at D5. Under salt stress, CAT and proline attained maximum concentrations at D3, MDA reached its highest level at D4, while POD exhibited continuous elevation through D5. Proline levels decreased at D4 but increased at D5 during salt exposure (**Figures 7A, B**).

Given the known importance of WRKY factors in stress resistance, 9 *LjWRKY* genes were selected from the 41 identified *LjWRKY* genes based on homologous sequence comparison with *A. thaliana* and *Oryza sativa* (**Table 1**) Specific primers for real-time quantitative PCR were designed to measure their activities (**Supplementary Table S1**) to verify the expression levels of these genes when plants are under stress. In unstressed *L. japonica* plants, the nine analyzed *LjWRKY* genes displayed constitutive organ-specific expression patterns. *LjWRKY36* showed predominant expression in roots, while *LjWRKY22* exhibited highest transcript levels in leaves. *LjWRKY10* and *LjWRKY31* were primarily expressed in stems, and *LjWRKY16* maintained comparable expression levels across roots, stems, and leaves (**Figure 7C**).

**Table 1:** WRKY transcription factors involved in plant drought and salt stress responses and their homologous sequences in the *LjWRKY* family.

Gene name	*Species*	Function	References	Related to *L. japonica*
*AtWRKY53*	*A. thaliana*	drought tolerance	(Sun and Yu, 2015)	*LjWKKY13,17,29,41*
*AtWRKY57*	*A. thaliana*	drought tolerance	(Jiang et al., 2012)	*LjWKRY16,22*
*OsWRKY54*	*O. sativa*	salt tolerance	(Huang et al., 2022)	*LjWRKY10,31*
*OsWRKY28*	*O. sativa*	salt tolerance	(Zhang et al., 2023)	*LjWRKY36*

### 3.7 Response of *LjWRKY* genes to drought and salt stress

**Figure 8:**
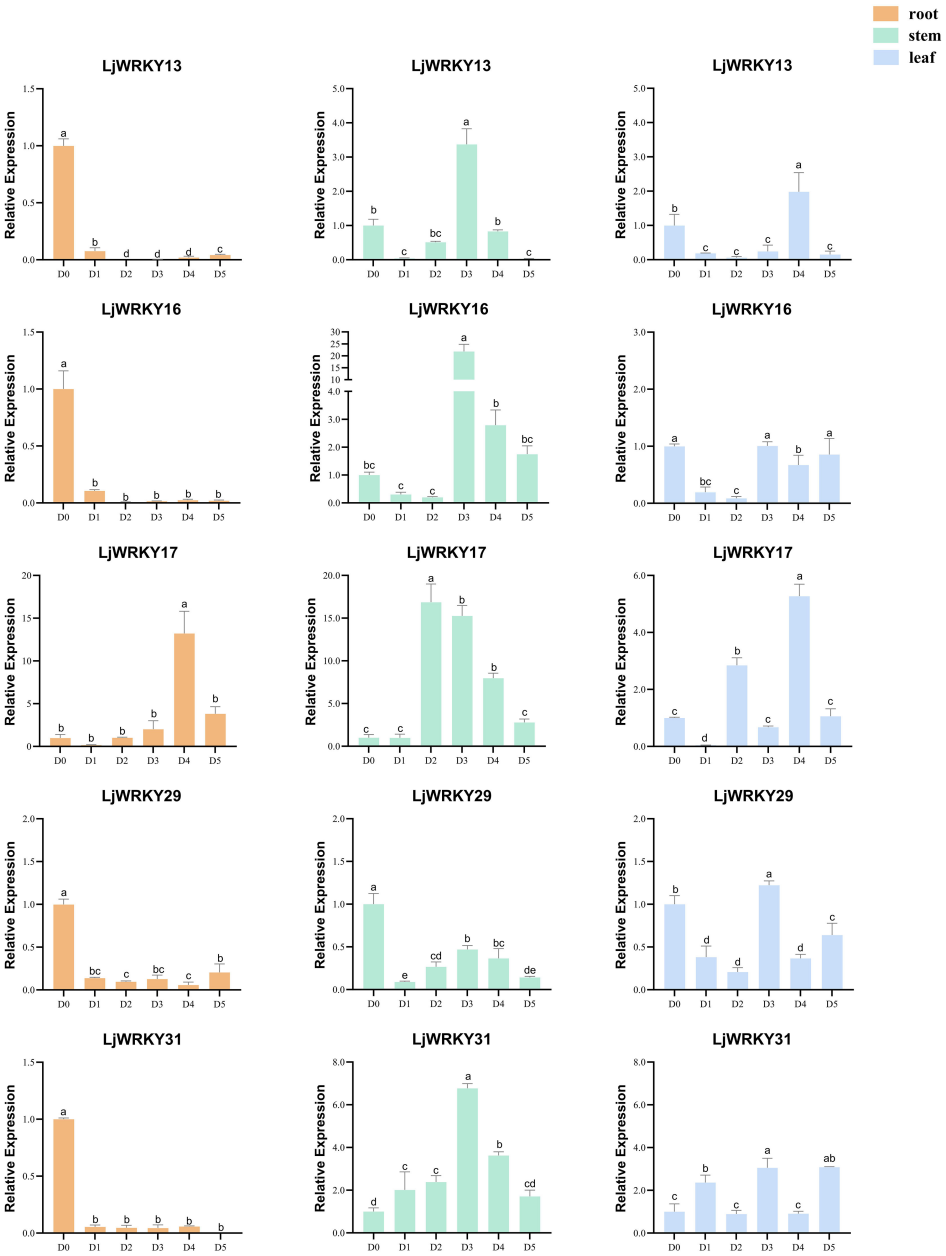
Expression profiles of some *LjWRKYs* under drought treatment determined by RT-qPCR. D0 (Control, well-watered); D1-D5 (Days 21–25 post-watering withdrawal). Bars labeled with different lowercase letters a-f indicate statistically distinct values (p< 0.05; “a” = highest value). Full dataset for all nine genes is available in **Supplementary Figure S1**.

**Figure 9:**
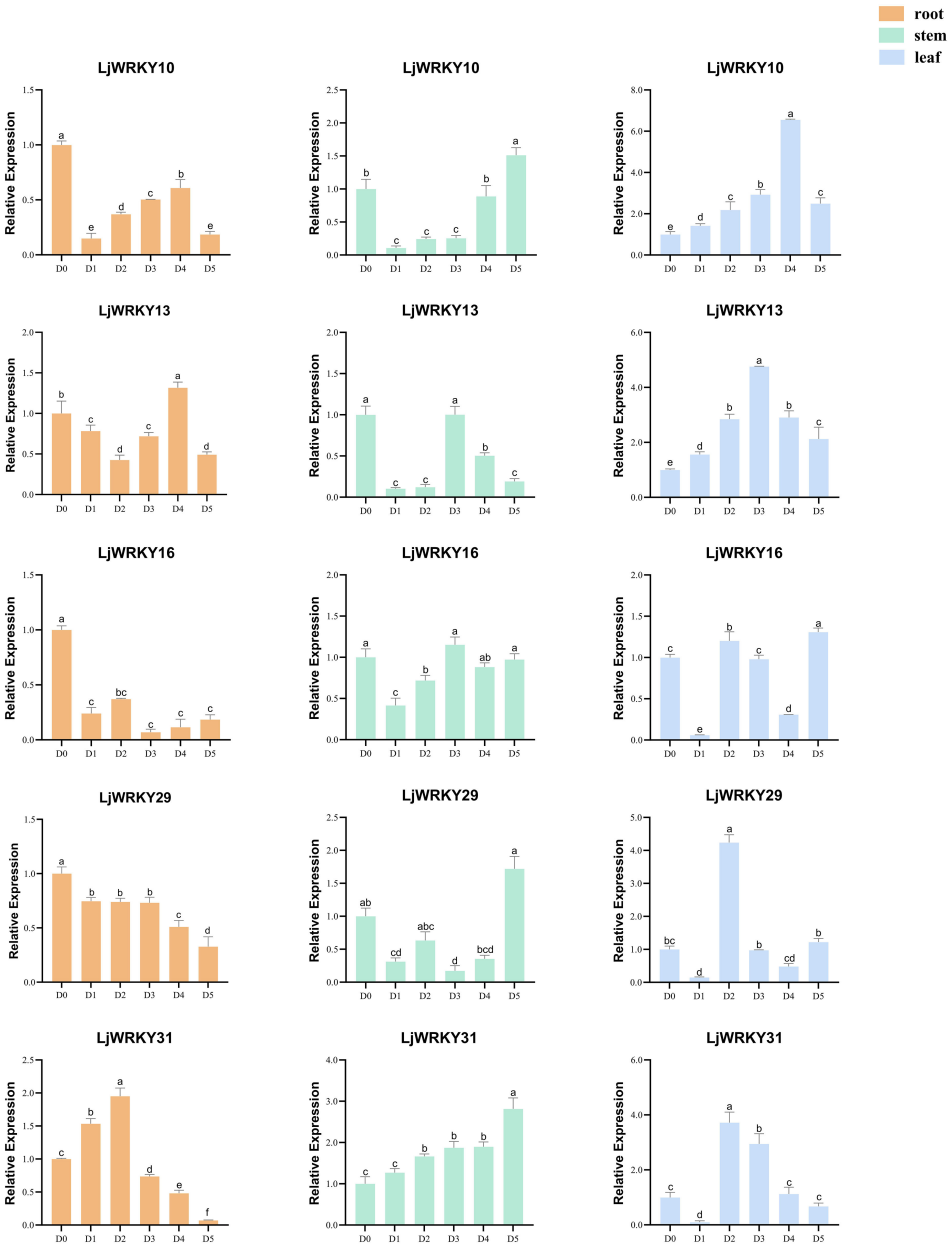
Expression profiles of some *LjWRKYs* under salt treatment determined by RT-qPCR. D0 (Control, untreated); D1-D5 (Days 1–5 of consecutive NaCl treatment). Bars labeled with different lowercase letters a-f indicate statistically distinct values (p< 0.05; “a” = highest value). Full dataset for all nine genes is available in **Supplementary Figure S2**.

Under drought and salt stress conditions, the nine analyzed *LjWRKY* genes exhibited distinct temporal and spatial expression patterns. During early drought stages (D1-D2), reduced expression levels of *LjWRKY13*, *LjWRKY16*, *LjWRKY29*, and *LjWRKY31* were observed in root tissues. Most genes reached peak expression during mid-stress periods (D3-D4). Specifically under drought conditions at D4, *LjWRKY22* expression increased in leaves, while under salt stress at D4, *LjWRKY10* expression increased in stems. During salt exposure, *LjWRKY31* showed progressively elevated expression in stems across time points, *LjWRKY16* exhibited stress-specific induction patterns distinct from controls, and both *LjWRKY10* and *LjWRKY31* displayed increased expression at D5 (**Figures 8**, **9**).

### 3.8 Correlation between physiological indicators and *LjWRKY* gene expression levels

**Figure 10:**
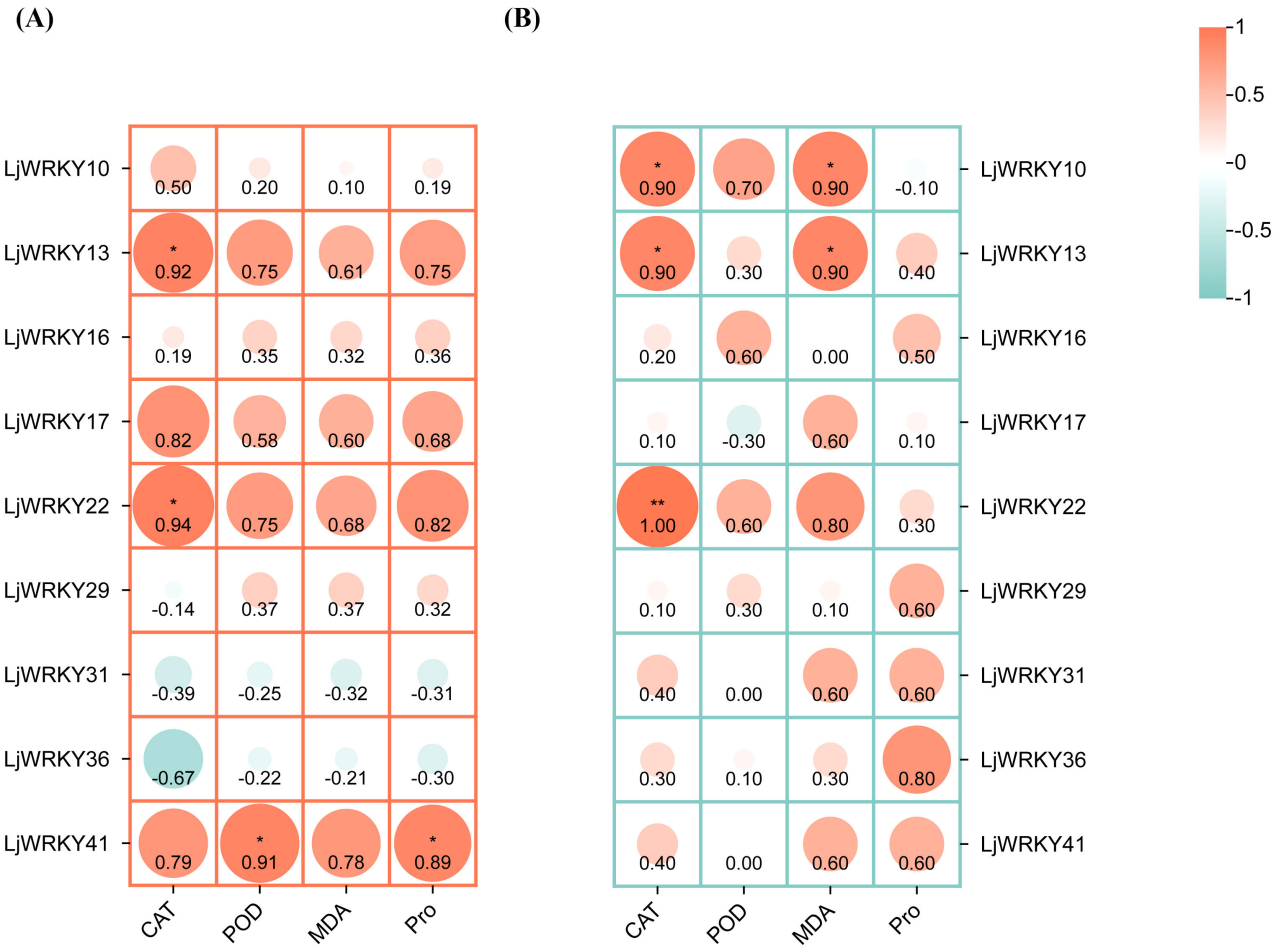
Correlation heat map between physiological indicators and *LjWRKY* genes of *L. japonica* under drought **(A)** and salt **(B)** stress. *Indicates significant correlation, p < 0.05; **indicates extremely significant correlation, p < 0.01.

To explore the correlation between the activities of these 41 *LjWRKY* genes and the physiological responses of plants under stress, this study used the Spearman statistical method for correlation analysis. This analysis helps to understand whether there is a connection between the level changes of four enzymes and compounds and the activities of nine specific *LjWRKY* genes during drought and salt stress (**Figure 10**). When these plants are subjected to drought, a total of four important relationships are found. *LjWRKY41* is clearly related to changes in peroxidase and proline, indicating that it plays a role in the plant’s response to water shortage. Similarly, *LjWRKY13* and *LjWRKY22* are significantly related to changes in catalase activity. Catalase is also an enzyme involved in plant stress responses. Under salt stress, a total of five genes with significant relationships are identified. *LjWRKY22* is closely related to changes in catalase activity. In addition, *LjWRKY10 and LjWRKY13* are related to changes in catalase activity and the level of propylene glycol (a compound indicating cellular stress).

## 4 Discussion

Increasing evidence indicates that *WRKY* is widely involved in various physiological and biological processes, including plant growth and responses to abiotic stresses (Jiang et al., 2017). Honeysuckle is a widely used traditional Chinese medicinal material. Due to its rich chlorogenic acid content, it is a species with significant economic and medicinal value. The completion of genome sequencing for this species provides a good opportunity for studying the gene families of *L. japonica*, which in turn will aid in the breeding and improvement of *L. japonica*. In this paper, relevant bioinformatics analyses such as phylogeny, conserved motifs and conserved domains, prediction of cis-acting elements, chromosome localization, and collinearity analysis of *L. japonica* were conducted, laying an important foundation for further research on *L. japonica WRKY* genes and enhancing the stress resistance of *L. japonica*. In this study, a total of 41 *LjWRKY* genes were found and can be divided into three different groups. The proportions of *WRKY* genes represented by each group vary; the proportion of *WRKY* genes in the second group is the highest, accounting for 63.41%. At the same time, the genetic distance between groups II and III is closer, which may be because group III originated from group II through the duplication of *WRKY* genes. Subcellular localization prediction shows that only *LjWRKY28* is located in the chloroplast, while other *LjWRKYs* are located in the nucleus, suggesting that *LjWRKY28* may have different functions.

The analysis of conserved motifs and phylogenetic trees of LjWRKY protein sequences reveals obvious group specificity. For example, LjWRKY28 and LjWRKY12 have two WRKYGQK motifs and belong to group I, but in the phylogenetic tree, they are closer to group II. Motif analysis shows that some motifs are unique to groups I and III, which may lead to the specificity of gene functions in these two groups. Compared with other groups, groups IIe and III only include two conserved motifs. On the other hand, some changes in gene structure, including point mutations in the coding DNA sequence region and regulatory sites of repeated members, affect the functions of new members. For example, LjWRKY3 loses the zinc finger structure sequence at the C-terminus, which may imply that LjWRKY3 has some new functions. Gene co-linearity relationship and gene structure diagram were combined for analysis, Group I/II genes respond to acute stress via hub interaction networks, whereas Group III members (e.g., *LjWRKY31*) utilize 5’UTR-mediated mRNA stability regulation to maintain metabolic balance under sustained stress. This complementary strategy provides new approaches for crop resistance breeding: developing hub-enhanced varieties for sudden disasters and creating UTR-modified cultivars for chronic climate challenges.

Cis-acting elements play an important role in gene transcription and expression. This paper analyzed and found a large number of light-responsive elements (G-box, GT1-motif, and Sp1), MeJA-responsive elements (TGACG motif and CGMCA motif), abscisic acid-responsive elements (ABRE), low-temperature-responsive elements (LTR), auxin-responsive elements (TGA-element and RST RR-core), and gibberellin-responsive elements (TATC-box). This indicates that *L. japonica WRKY* genes are widely involved in transcriptional regulation processes such as growth and development, defense and stress response, and secondary metabolism. This is basically consistent with the analysis results of cis-acting elements of most plant *WRKY* gene family members (Abdullah-Zawawi et al., 2021). Light-responsive elements are one of the most abundant elements in *L. japonica*, and the second is MeJA elements. Exogenous MeJA induces *WRKY* genes containing MeJA elements and may enhance *L. japonica*, thereby participating in the regulation of other plant physiological processes. WRKY transcription factors can specifically bind to the W-box element in the gene promoter and affect gene transcription (Chen et al., 2019).

Duplicated genes may exhibit different expression patterns. Gene duplication and polyploidy in plants also appear to extend to gene family members. Based on chromosomal localization and gene cluster analysis, the expansion of the *LjWRKY* gene family is primarily co-driven by two mechanisms: (1) Tandem duplication (exemplified by the closely linked *LjWRKY31-LjWRKY32* gene pair with no intervening genes on chromosome 8), which rapidly generates adaptive genetic variation through local replication; and (2) Dispersed duplication (where some genes are independently scattered), potentially forming new genes with regulatory functional differentiation via transposition. These two mechanisms act synergistically to enhance the functional diversity and evolutionary adaptability of this family in stress responses. This study analyzed the genomic syntenic relationships between *L. japonica* and *A. thaliana*, *Hylocereus undatus*, and *L. macranthoides*. In these three comparative analyses, 33, 35, and 41 pairs of *WRKY* homologous gene pairs derived from segmental duplication were identified between *L. japonica* and the other three species, respectively. Notably, all *WRKY* genes from *L. japonica* could be mapped to duplicated segments in its congeneric species *L. macranthoides*. This indicates that the *WRKY* gene family in *L. japonica* is relatively evolutionarily conserved, and its functional and structural characteristics are likely highly similar to those in other species within the genus. Further analysis revealed that among the syntenic gene pairs between *L. japonica* and the other three species, 70 gene pairs were subjected to strong purifying selection during evolution. Particularly striking was the high sequence conservation observed in five of these gene pairs, all of which existed in the syntenic gene pairs between *L. japonica* and *L. macranthoides*. These results collectively confirm the conserved nature of the *LjWRKY* gene family.

Under stress conditions, the production and accumulation of ROS (reactive oxygen species) are highly destructive to macromolecules and cell integrity, eventually leading to cell death (Ojcius et al., 2010; Marchi et al., 2012). The evaluation of reactive oxygen species triggers the cellular antioxidant system and is an important pathway for cellular physiology and biochemistry (Theocharis et al., 2012). Plants protect themselves by activating the reactive oxygen species scavenging enzyme system under this threat. An effective reactive oxygen species scavenging enzyme system includes peroxidase (POD) and catalase (CAT) (Jiang et al., 2013). Malondialdehyde (MDA) is a product of the peroxidation of unsaturated fatty acids and may play an important role in destroying cell membranes (Prasad et al., 2019). Proline is an organic osmolyte that accumulates in various plants under stress conditions, stabilizes membranes, thereby preventing electrolyte leakage and scavenging ROS (Verbruggen and Hermans, 2008; Hayat et al., 2012). As the drought time prolongs, the content of various physiological indexes of honeysuckle leaves shows a trend of first increasing and then decreasing (**Figure 7A**), indicating that plants have a positive physiological response at the initial stage of stress. By improving catalase and peroxidase activity, MDA and proline content, the antioxidant defense mechanism of honeysuckle is improved to resist oxidative stress caused by drought. However, as the stress time prolongs and the degree of drought increases, the plant damage increases significantly, and the drought resistance gradually weakens, and the activities of catalase and peroxidase also decrease. In this experiment, as the salt concentration increases, the CAT activity of honeysuckle leaves shows a trend of first increasing and then decreasing (**Figure 7B**). At low concentrations, the content of reactive oxygen species in plants gradually increases, and the enzyme activity of scavenging active substances also increases. As the salt concentration increases, the content of reactive oxygen species in plants reaches a certain level, causing damage to plant cells, resulting in the inability to produce more antioxidant enzymes, so the antioxidant enzyme activity shows a downward trend (Hernández et al., 2006; Miller et al., 2010).

WRKY transcription factors are crucial for controlling the plant’s response to stress. Studies have shown that the *A. thaliana* WRKY57 transcription factor may confer drought tolerance to transgenic rice plants. Overexpression of *AtWRKY57* in rice improves the tolerance of rice to drought, salinity, and polyethylene glycol (PEG) (Jiang et al., 2016). The novel *MxWRKY53/64* gene isolated from *Matthiola incana* is a nuclear-localized protein, and its expression level is strongly affected by salt and iron. When *MxWRKY53/64* is introduced into transgenic *A. thaliana*, the resistance to salt and iron stress is significantly increased (Han et al., 2021). This study shows that nine *WRKY* genes are widely expressed in the roots, stems, and leaves of *L. japonica*, and the expression of nine *LjWRKY* genes has obvious tissue specificity, indicating that *WRKY* genes may play an important role in the process of plant growth and development (**Figure 7C**). *LjWRKY13, 16, 19*, and *21* are inhibited under drought stress (**Figure 8**), indicating that these genes are sensitive to drought, which may mean that these genes or some upstream genes play an important role in the drought tolerance regulatory network of *L. japonica*. Under salt stress, with the prolongation of stress time, the expression level of *LjWRKY31* in stems gradually increases (**Figure 9**), indicating that the high expression of *LjWRKY31* may contribute to the enhancement of salt tolerance of *L. japonica*. The gene structure of *LjWRKY31* shows a UTR at its 5’ end, which likely mediates mRNA stability to maintain metabolic balance under prolonged stress (**Figure 3C**). *LjWRKY31* may be a key gene in the salt tolerance regulatory network of *L. japonica*. In addition, the correlation between *LjWRKY22* gene expression and physiological indicators also shows that these genes may change the drought tolerance and salt tolerance of *L. japonica* by regulating certain reactive oxygen scavenging enzymes. Especially under drought stress and salt stress, the correlation between *LjWRKY22* gene expression and CAT content in leaves is very significant (**Figure 10**).

**Figure 11:**
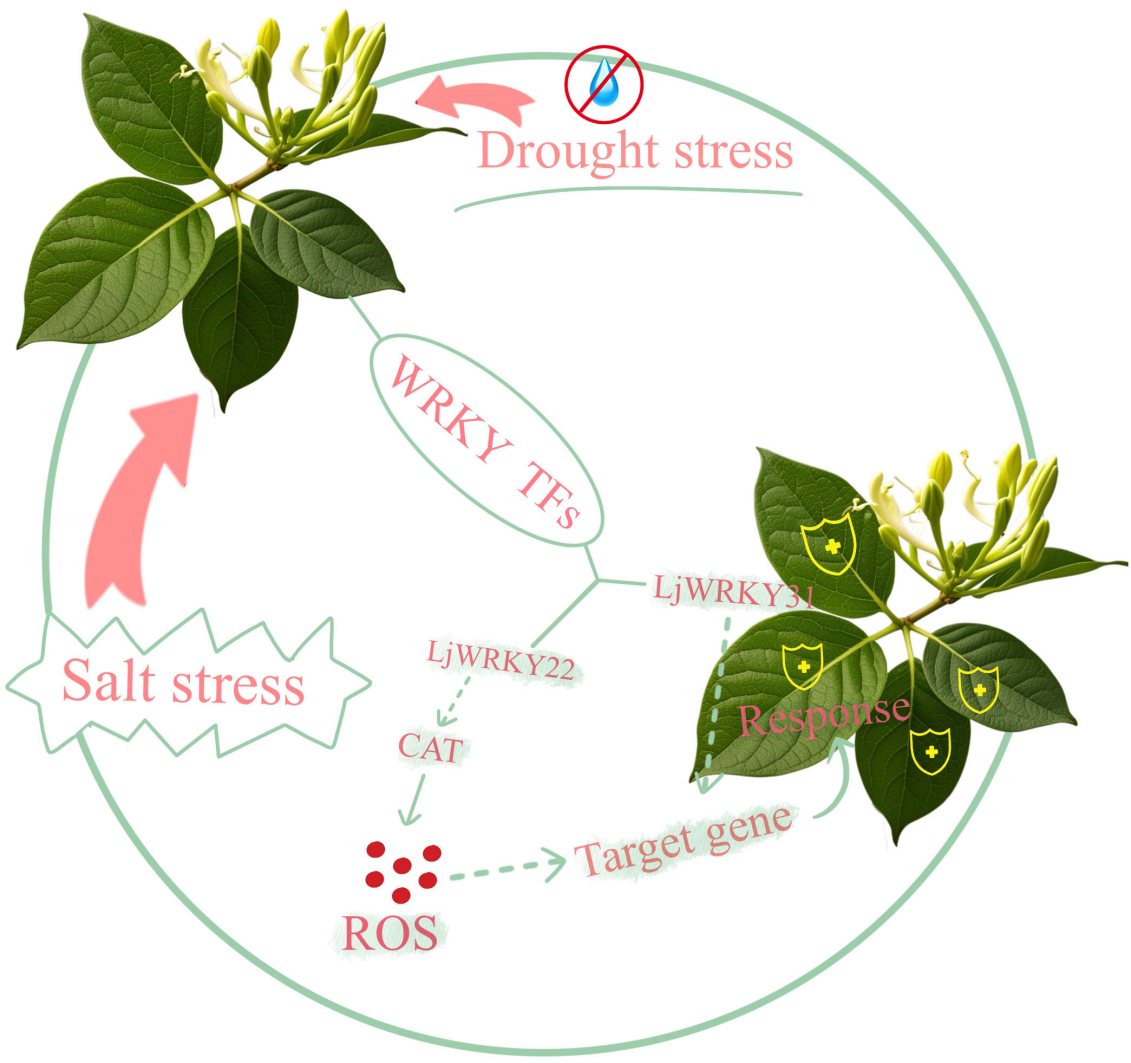
Mechanism diagram of *LjWRKY* family under drought stress and salt stress.

The *WRKY* regulatory network (**Figure 11**) elucidates *Lonicera japonica WRKY*-mediated stress adaptation mechanisms, demonstrating how *LjWRKY22* activates CAT biosynthesis to maintain ROS homeostasis during salt stress, while *LjWRKY31* coordinate drought and salt responses through its respective target genes. Although this framework integrates core signaling pathways, it does not encompass cross-regulatory dynamics with other transcription factor families (e.g., NAC, bZIP), epigenetic regulation of *LjWRKYs*, or protein interaction networks - limitations necessitating future investigation through spatially resolved techniques such as single-cell omics.

In conclusion, through systematic research and expression pattern analysis, this study identified multiple genes closely related to the stress response of *L. japonica*, but their specific functions and interactions still need to be verified. In the future, further functional verification and interaction analysis of these genes are needed to better understand the key roles and mechanisms of the *WRKY* gene family under abiotic stresses.

## 5 Conclusion

This study conducted in-depth research on the *WRKY* gene family of *L. japonica*. 41 full-length *WRKY* genes were described and further divided into three main categories, with extremely similar motif compositions in the same groups and subgroups. Homology analysis and phylogenetic comparison of *WRKY* genes in different plants help understand the evolutionary characteristics of *L. japonica WRKY* genes. 9 *LjWRKY* genes play an important role in the response of *L. japonica* to drought and salt stress. The expression patterns of these genes in different tissues and their responses to salt stress and drought treatment have been confirmed. In addition, *LjWRKY22* may improve the drought tolerance of plants by regulating the content of catalase in the roots of *L. japonica*. *LjWRKY31* may be a potential target gene for improving the salt tolerance of *L. japonica* through biotechnology or molecular breeding. Finally, we obtained the working model of the *WRKY* gene family in *L. japonica*. These data provide important resources for an in-depth understanding of the biological functions of specific *WRKY* genes of *L. japonica*.

## Data Availability

The datasets presented in this study can be found in online repositories. The names of the repository/repositories and accession number(s) can be found in the article/**Supplementary Material**.

## Supplementary material

Supplementary Table 1 The primers used in this study.

Supplementary Table 2 CDS and protein sequences of LjWRKYs.

Supplementary Table 3 Physicochemical characteristics and subcellular localization of *LjWRKYs.*

Supplementary Table 4
*LjWRKYs* promoter cis-acting elements.

Supplementary Table 5 Collinear associations between L. japonica and three species.

Supplementary Figure 1 Expression profiles of 9 *LjWRKYs* under drought stress.

Supplementary Figure 2 Expression profiles of 9 *LjWRKYs* under salt stress.
